# Supplementary material for: Integrating genomic structural equation modeling and experimental validation to unravel the genetic basis of male genital lichen sclerosus
Source: Front Immunol. 2026 Jul 16;17:1857945. doi: 10.3389/fimmu.2026.1857945 (PMC13435250; doi:10.3389/fimmu.2026.1857945)

Genomic Region: 7:126869221-7:128778386

1st Population

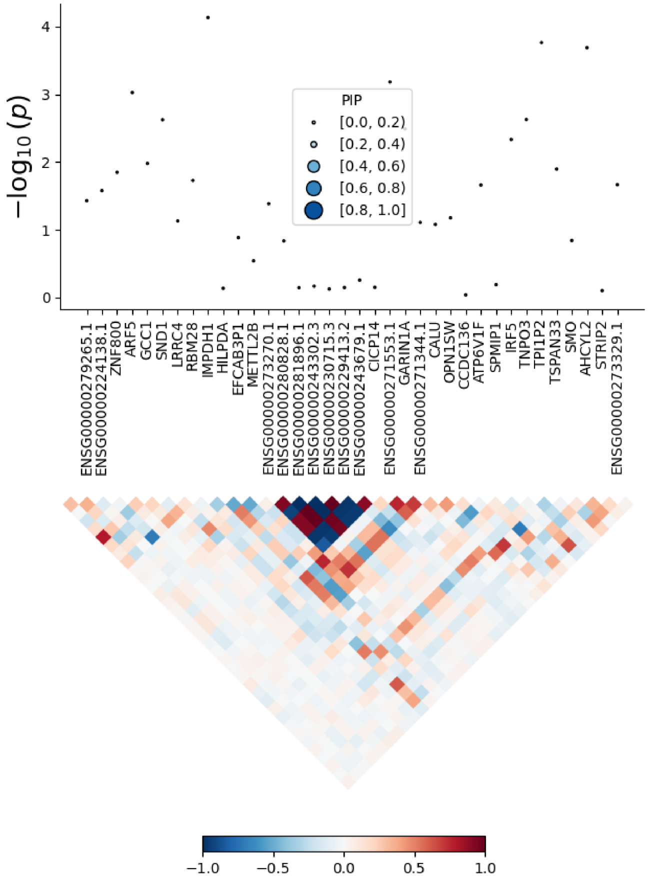

Supplement: Supplementary file 6 [file SupplementaryFile5.zip › result/fuma_PSYCH_factor_sCCA3_focus.chr7.chr7.126869221.128778386.pop1.pdf]
